# Virtual reality for training emergency medicine residents in emergency scenarios: usefulness of a tutorial to enhance the simulation experience

**DOI:** 10.3389/fdgth.2025.1466866

**Published:** 2025-02-18

**Authors:** A. Vittadello, S. Savino, S. Bressan, M. Costa, A. Boscolo, N. Sella, T. Pettenuzzo, F. Zarantonello, A. De Cassai, T. Chang, P. Navalesi, G. Mormando

**Affiliations:** ^1^ Department of Medicine (DIMED), University of Padua, Padua, Italy; ^2^ UOC Accettazione e Pronto Soccorso, Azienda Ospedale-Università Padova, Padua, Italy; ^3^ Department of Women’s and Children’s Health, University of Padua, Padua, Italy; ^4^ Institute of Anaesthesia and Intensive Care, Padua University Hospital, Padua, Italy; ^5^ UOC Istituto di Anestesia e Rianimazione, Azienda Ospedale Università, Padua, Italy; ^6^ Keck School of Medicine, University of Southern California, Los Angeles, CA, United States; ^7^ Division of Emergency and Transport Medicine, Children’s Hospital Los Angeles, Los Angeles, CA, United States

**Keywords:** virtual reality, simulation, familiarization, emergency medicine training, tutorial

## Abstract

**Introduction:**

Critical events in healthcare require a rapid and coordinated approach: simulation has been demonstrated a valid technique for training in emergency. Virtual Reality (VR) is an innovative technology that has revolutionized simulation training and healthcare professional development. A key phase of a simulation session with manikin consists in a familiarization with setting and equipment. The primary objective of this study is to investigate whether familiarization with a VR tutorial can change the perception of cases.

**Methods:**

Emergency medicine residents were randomly assigned to the Intervention group (n = 21) who undergone familiarization tutorial prior to the clinical scenario to a Control group (*n* = 21) where no familiarization tutorial was provided before the clinical scenario.

**Results:**

No significant differences were found between the two groups regarding perceived ease of use, but the Intervention group found VR familiarization useful and the Control group found it necessary to implement a VR tutorial. VR training was generally perceived by learners as a useful technology for training as confirmed by the literature.

**Discussion:**

Familiarization seems to be an important phase of simulation-based training for trainees, even when running a VR-based simulation for an emergency scenario; it should be incorporated into the clinical VR sessions for simulation in healthcare settings.

## Background

Emergency medicine is a clinical field that requires a combination of clinical expertise with rapid decision-making capability in contexts that can often be stressful. In these critical and complex settings, time is a key parameter (1). The management of the critical patient requires a rapid and coordinated approach, with the aim of stabilizing the patient, identifying and treating the underlying cause.

Therefore training is essential to learn and improve compliance to stabilization protocols and decision-making. Simulation has often been used for training in healthcare and emergency settings as it allows the opportunity to train by experiencing challenging critical scenarios while increasing patient safety (2, 3). Some studies suggest that simulation-based training in healthcare is superior to training that does not use this tool even though it takes longer to implement (4).

The use of virtual reality (VR) in medical simulation has revolutionized training and professional development. Virtual reality offers trainers immersive experiences that transport them into simulated environments, allowing them to interact and learn in realistic virtual contexts. This technology is widely used for training in the medical area and especially in emergency care due to its ease of implementation and the opportunity to repeat the clinical scenario in an immersive, realistic, and safe environment. A meta-analysis has shown that this teaching modality has a positive impact on educational outcomes in all learning environments (5). In the healthcare field, numerous studies have shown a positive effect of using VR on learning (6–9). VR sessions had a good perceived usefulness and led to an increase in the perceived level of competence in undergraduate students undergoing VR-based training in emergency scenarios (10).

Information provided prior to the simulation experience is fundamental to the success of the simulation and can enhance the effectiveness of the simulation-based experience (11). One of the key criteria for conducting a simulation training session is to provide knowledge on the use of equipment and orientate learners to all factors involved in the simulation itself including equipment, scenario setting or other technological environments (12). The tutorial, prior to the use of new software or hardware, should lead to a reduction in the cognitive load of the simulation participant who can focus on the clinical case by having the tools to handle the technology present in the simulation. Many simulation softwares in the medical field do not have a tutorial within them to provide the learner with all the basic knowledge to cope with the simulation. According to cognitive load theory, information processing capacity is limited. Learning can be inhibited if the learning situation exceeds the cognitive capacity of the learner (13).

Although the literature agrees that information overload is detrimental to learning (14, 15), there are no data on the effect of orientation and familiarization in VR-based medical simulation. Our primary outcome is to study whether a tutorial familiarization in addition to a VR session can modify the scenario's perception.

## Methods

This is a single-center, randomized (1:1) trial conducted at the University of Padova, Italy in 2024 comparing a VR emergency simulation scenario delivered with and without a VR familiarization tutorial. The study took place at the “SIMULARTI” Medical Simulation Center of Medicine Department (DIMED) of the University of Padova, Padova, Italy, between April and May 2024.

The authors confirm that this study was performed in accordance with the ethical standards as laid down in the 1964 Declaration of Helsinki and its later amendments.

The study reports the extension to the CONSORT statement for healthcare simulation research (see Supplementary Table 1, Supplemental Digital Content 1—Checklist: Simulation-based Research Extensions for the CONSORT Statement) (16).

The participants did not receive any incentives and they provided written consent before participating. All data were collected anonymously. Individual participants were randomly allocated an ID number, which was only known to participants, in order to calculate the perceived level of competence before and after the VR sessions and the perceived usefulness of a tutorial to be performed before the simulation.

Emergency medicine (EM) residents of the University of Padua (Padua, Italy)—which underwent standardized training during simulation-based courses during residency in addition to regular traditional teaching- were recruited by email. Basics of neurologic emergencies were previously delivered as lectures during the residency program as part of the syllabus, but neither practical training nor functional exercises were performed before the study.

**Figure 1:**
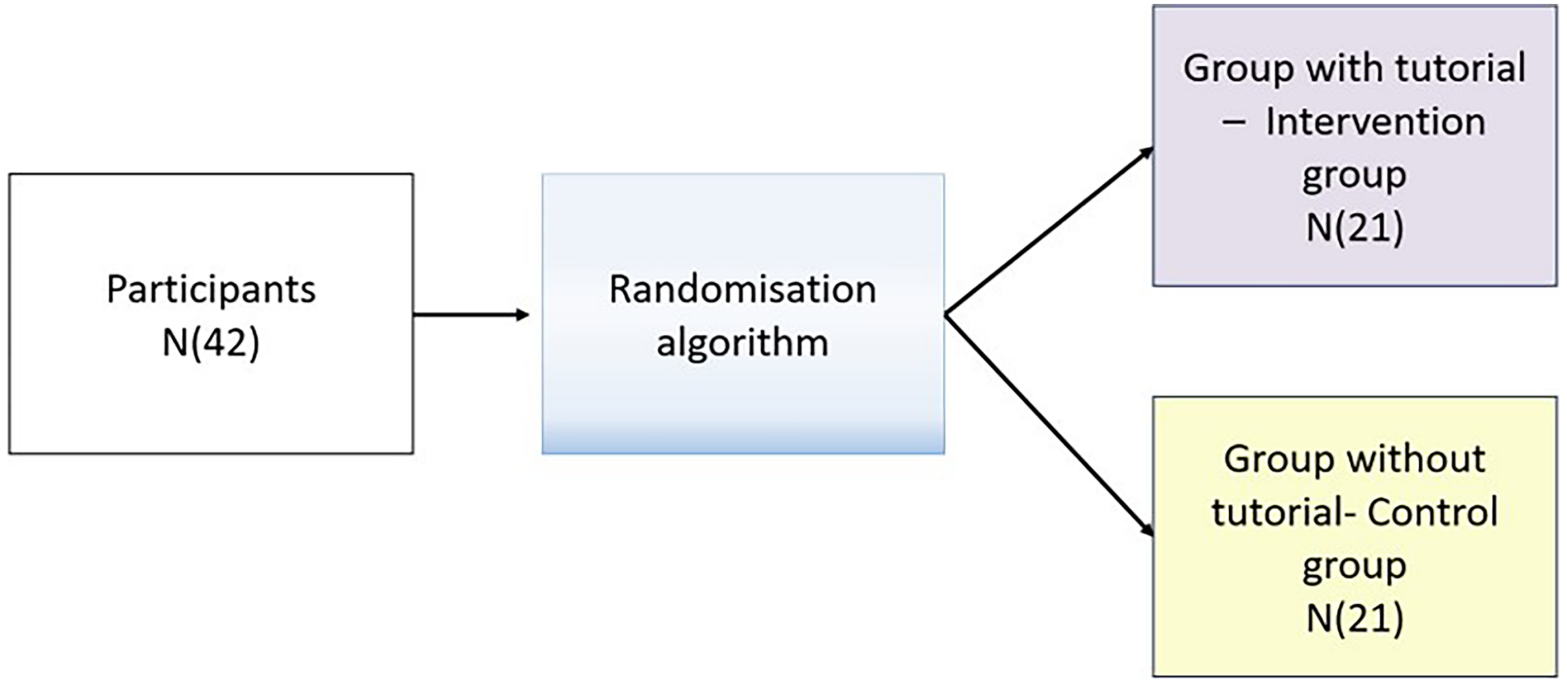
Randomisation diagram.

For the 1:1 allocation, a computer-generated list of random numbers was used. Participants were randomly assigned to either the Intervention group (VR scenario delivered with a familiarization tutorial preceding the scenario) or a Control group (VR scenario delivered without a familiarization tutorial preceding the scenario) (Figure 1). Randomization and blinding did not directly involve the researchers of the study.

All the participants had to manage one single scenario of status epilepticus on a female adult patient. The scenario is one of those available on the VR software Resuscitation VR Italian version (v0.63—i3 Simulations, London, UK) and it was delivered using Oculus Quest 2 headsets (Meta, San Jose, CA, USA). There was no time limit imposed neither for the scenario nor for the tutorial, that each participant could run at his/her own pace. The tutorial and the scenario both took around 10 min each to complete (precise measurement of the elapsed time was not recorded as it was out of the scope of the study). The Control group performed the scenario without any VR familiarization tutorial; the Intervention group, before the scenario, underwent one tutorial to get acquainted with the VR software. The tutorial used was the one provided in the Resuscitation VR software, and developed by i3 Simulations (London, UK). The tutorial involved the trainee being immersed in the simulation room in which he would later perform the real scenario, and the technical equipment, material and characters were replaced with rubber duckies. In this way, the learner could understand how to use the VR controllers and interact with the equipment and drugs in the medical scenario they would later immerse themselves in for the clinical case.

### Outcome measures

The anonymous questionnaire “Technology Assessment Questionnaire to evaluate the perceived usefulness and perceived ease-of-use” (Supplementary Table 1) was administered to the participants to evaluate their perception of the experience using a likert- 7 scale, from “very low” to “very high.” Residents were also asked to rate their *perceived level of competence* in managing a clinical scenario before and after the VR session on a scale, from “very low” to “very high.” Using the same methodology, the participants' perception of the need for a tutorial in approaching VR was analyzed.

Information on participant demographic, experience with simulation and with videogames, was also collected via an electronic survey.

## Results

42 EM residents participated in the study, aged 24–50 years old (median = 28 years; IQR range = 26.3 −30.0 years old). Most were EM residents in their first year of residency (22, 52%) and prevalently female (27, 64%). The experience with video games was similar in the two groups with a median of 2 (IQR range 1–4), their experience with simulation was also comparable. All participants had an American Heart Association (AHA) certification in their curriculum, within which there is a practical part on a mannequin for the certification itself (Table 1).

**Table 1:** Characteristics of populations.

*N°*		Intervention group	Control group
		21	21
Sex	M	6 (29%)	9 (43%)
	F	15 (72%)	12 (57%)
Age	Median (IQR range)	28 (26–32)	28 (27–29
Current year of residency	Median (IQR range)	1 (1–3)	1 (1–3)
How do you rate your experience with video games (likert scale 1–7)	Median (IQR range)	2 (1–4)	2 (1–5)
How do you rate your experience with VR before this project? (likert scale 1–7)	Median (IQR range)	1 (1–2)	1 (1–2)

The perceived usefulness assessed as the median adjusted to the cent was 83/100 [interquartile range (IQR) 50–100].

We evaluated the perceived ease of use with the following questions: “Learning how to use Resuscitation VR was easy for me.”, “It was easy to make Resuscitation VR do what I wanted it to do.”, “The interaction with Resuscitation VR was clear and understandable”. “It was easy for me to become proficient in the use of Resuscitation VR.” “I found Resuscitation VR easy to use” (Supplementary Table 1). As shown in Table 2, no significant differences were found between the Intervention group and the Control group. However, the Intervention group (median adjusted to the cent 67/100 IQR 33–83) showed a better response to the ease of use during the Resuscitation VR scenario than the Control group (median 50/100 IQR 16–67).

**Table 2:** Characteristics of the answers to the perceived ease-of-use questions of the technology assessment questionnaire.

Question	Intervention group		Control group		*U* di Mann–Whitney
	Median	IQR range	Median	IQR range	*p*- value
Learning how to use resuscitation VR was easy for me	5	4–6	4	4–6	0.247
It was easy to make resuscitation VR do what I wanted it to do	5	2–3	3	2–3	0.291
The interaction with resuscitation VR was clear and understandable	5	3–6	4	2–5	0.072
It was easy for me to become proficient in the use of resuscitation VR	5	3–5	3	2–3	0.077
I found resuscitation VR easy to use	5	4–6	4	3–6	0.192

**Figure 2:**
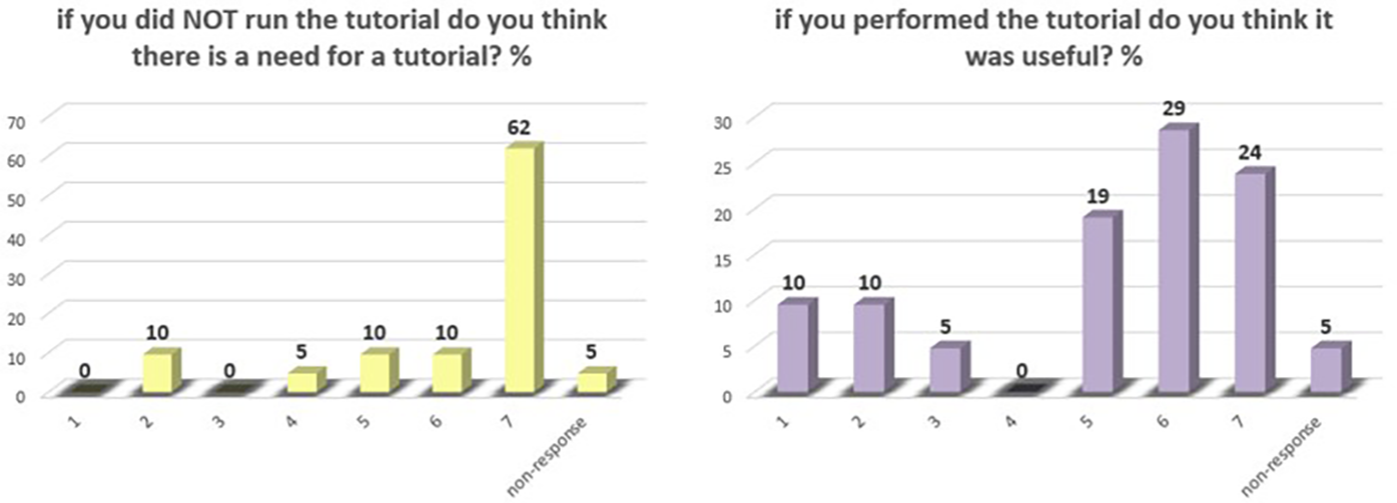
Bar graph on the responses of the perceived need for a tutorial in the approach to VR on a Likert scale from 1 to 7 in percent.

In the final analysis, we asked the Intervention group using a likert scale of from 1 to 7 whether they found it useful, obtaining a median response of 6 (IQR range 4.50–6.25). Similarly, we asked the Control group if they considered the implementation of a tutorial necessary and the median of the answers was 7 (IQR range 5.75–7.0). In this last question there was a general agreement on the usefulness of the tutorial, with no participant answered with a value of 1 (Figure 2).

## Discussion

VR training was generally perceived by learners as a useful tool for training. This perception is also confirmed in the literature, present in several other articles (10) or in the review by Abbas et al. (17); in fact, VR was described as usable, satisfying and in some cases preferred over traditional educational method.

The two study groups, the Intervention and the Control group, were homogeneous (Table 1), both the participants' experience with VR and the training experience taken into consideration with the year of the residency course as well as the external training assessed with accredited courses were completely comparable in the two groups.

We did not find any difference between perceived usefulness of VR in the two study groups probably because VR guarantees such immersiveness that the residents perceived it an useful training independently of the exposure to a familiarization tutorial. The young average age of the participants, which implies a generational advantage with the technological equipment present, may have play a role in the results obtained with respect to perceived usefulness and ease of use (18).

There was considerable consensus on the usefulness of the tutorial itself as an approach prior to the start of the simulation session. On the other hand, the majority of the trainees who had not performed any tutorials prior to the scenario considered the presence of a familiarization in the VR environment to be appropriate. This concept is in line with the best practice standards of simulation in medicine (12), leading to the need to best define familiarization procedures and needs for all technologies that are to be used in simulation. This concept then becomes of considerable importance when referring to the numerous different softwares that are populating today's commercial market. It is believed that this gives rise to the need for each manufacturer to opt for a tutorial based on the simulation environment that the learner will be engaged with.

## Limitations and future prospects

Our work has some limitations related to its pilot design. The first limitation is the location in a single center. Second is the small sample size and the use of a single clinical topic and software. Finally, another limitation is not having data on the timing and performance of the scenario. Further studies that also consider clinical performance are needed to better define the best familiarization standards when using VR for medical simulation. Standardization of a familiarization tutorial is critical. At the present time, interfaces and technologies vary widely, which implies that it will be necessary to have a tutorial for each software. Guidelines will have to be created with future data in order to standardize familiarization with VR.

## Conclusion

Familiarization in a simulation performed in virtual reality seems to be an important part of education for trainees. The presence of the tutorial was considered a necessity by those who had not performed it. The introduction of notions for using the technology was considered useful when running a VR-based simulation for emergency scenarios. It should therefore be incorporated into VR clinical simulations. Further studies are needed to define the best familiarization standards when using VR for medical simulation.

## Data Availability

The raw data supporting the conclusions of this article will be made available by the authors, without undue reservation.
