# Supplementary material for: Virtual reality for training emergency medicine residents in emergency scenarios: usefulness of a tutorial to enhance the simulation experience
Source: Front Digit Health. 2025 Feb 18;7:1466866. doi: 10.3389/fdgth.2025.1466866 (PMC11876424; doi:10.3389/fdgth.2025.1466866)
Supplement: Supplementary file 1 [file Datasheet1.pdf]

Supplementary Material: Technology Assessment Questionnaire

	Extrem ely Unlikel y	Quite Unlikely	Slightly Unlikely	Neither	Slighlty Likely	Quite Likely	Extrem ely Likely
1. Using Resuscitation VR would enable me to learn more quickly							
2. Using Resuscitation VR would improve my learning performance							
3. Using Resuscitation VR would improve my learning engagement							
4. Using Resuscitation VR would improve my learning transfer							
5. Using Resuscitation VR would make it easier to learn							
6. I would find Resuscitation VR useful in my learning							
7. Learning to operate Resuscitation VR was easy for me							
8. I found it easy to get Resuscitation VR to do what I want it to do							
9. My interaction with Resuscitation VR was clear and understandable							
10. It was easy for me to become skillful at using Resuscitation VR							
11. I found Resuscitation VR easy to use							

Questionnaire scoring

Extremely Unlikely = 1, Extremely Likely = 7

Perceived Usefulness (PU) (out of 100) = (Average of Q1-6)
